# Thoracic epidural analgesia: a new approach for the treatment of acute pancreatitis?

**DOI:** 10.1186/s13054-016-1292-7

**Published:** 2016-05-04

**Authors:** Olivier Windisch, Claudia-Paula Heidegger, Raphaël Giraud, Philippe Morel, Léo Bühler

**Affiliations:** Department of Surgery, Geneva University Hospitals, Geneva, Switzerland; Division of Intensive Care, Geneva University Hospitals, Geneva, Switzerland

**Keywords:** Thoracic epidural analgesia/anesthesia, Acute pancreatitis, Mortality, Splanchnic perfusion, Microcirculation, Pancreas necrosis

## Abstract

This review article analyzes, through a nonsystematic approach, the pathophysiology of acute pancreatitis (AP) with a focus on the effects of thoracic epidural analgesia (TEA) on the disease. The benefit–risk balance is also discussed. AP has an overall mortality of 1 %, increasing to 30 % in its severe form. The systemic inflammation induces a strong activation of the sympathetic system, with a decrease in the blood flow supply to the gastrointestinal system that can lead to the development of pancreatic necrosis. The current treatment for severe AP is symptomatic and tries to correct the systemic inflammatory response syndrome or the multiorgan dysfunction. Besides the removal of gallstones in biliary pancreatitis, no satisfactory causal treatment exists. TEA is widely used, mainly for its analgesic effect. TEA also induces a targeted sympathectomy in the anesthetized region, which results in splanchnic vasodilatation and an improvement in local microcirculation. Increasing evidence shows benefits of TEA in animal AP: improved splanchnic and pancreatic perfusion, improved pancreatic microcirculation, reduced liver damage, and significantly reduced mortality. Until now, only few clinical studies have been performed on the use of TEA during AP with few available data regarding the effect of TEA on the splanchnic perfusion. Increasing evidence suggests that TEA is a safe procedure and could appear as a new treatment approach for human AP, based on the significant benefits observed in animal studies and safety of use for human. Further clinical studies are required to confirm the clinical benefits observed in animal studies.

## Background

Acute pancreatitis (AP) is one of the most frequent gastrointestinal diseases requiring hospital admissions worldwide. In the USA in 2009, AP was responsible for 275,000 hospital admissions with a cost of over US$2.5 billion [1]. The incidence of AP is also increasing due to an increase of risk factors such as obesity, contributing to the formation of gallstones, the main cause for development of AP [2]. Other frequent etiologies of pancreatitis are increased alcohol consumption and aging. The proportion of each cause varies among the studies, but gallstones appear to be the first cause of AP, representing 40–70 % of overall cases [3].

In 80 % of the cases the disease course is mild, but 20 % of patients will experience moderately-severe to severe disease. Despite an overall mortality of 1 %, the severe form of AP is associated with a mortality reaching 30 % [4]. New guidelines, based on evidence-based literature, emerge every few years and tend to reach consensus on the best clinical management of AP. Besides the endoscopic removal of gallstones if present, symptomatic treatment remains the cornerstone of medical therapy in AP. Symptomatic therapy focuses on aggressive rehydration, early nutrition, acceptable analgesia, oxygenation, and antibiotic use restricted to confirmed infections [4]. All of these approaches do not have a direct action on the pancreas itself but try to attenuate the process of the multiorgan dysfunction syndrome (MODS) present in the severe form of pancreatitis. No causal treatment has yet been developed.

Recent animal and human studies have analyzed the role of thoracic epidural analgesia (TEA) during AP. Their hypothesis is that selective segmental sympathectomy induced by TEA may improve splanchnic perfusion and induce changes in microcirculation, leading to a better perfusion of the pancreas [5].

The effect of TEA on splanchnic blood flow is an active domain of interest, with conflicting results. Richards et al. [6] published in 2013 a systemic review which could not establish the real impact of TEA on splanchnic perfusion. Siniscalchi et al. [7] also stated that studies focusing on regional macrocirculation could not show an influence on, or even showed worsening of, regional perfusion when TEA was used. In contrast, studies focusing on microcirculation, especially in pathologic flow conditions such as the state induced by AP, suggested that TEA could improve microcirculation [7]. There is growing evidence that microcirculation plays a crucial role in AP, and no treatment except TEA has proven to play a role in this aspect.

The goal of this article is to describe the pathophysiology of AP and to explore actual knowledge concerning the effect of TEA on AP by reviewing available animal and human studies. Furthermore we aim to evaluate the benefits and risks of TEA, discuss specific aspects relevant to its clinical application, and investigate its possible place in disease management.

### Pathophysiology of acute pancreatitis

The underlying mechanism of AP is a dysfunction of the pancreatic acinar cells, resulting from an inappropriate activation of trypsinogen, the proenzyme of trypsin, in its active form trypsin [8]. The presence of active trypsin in the pancreas activates trypsinogen in the gland, leading to an accumulation of trypsin in the acinar cells followed by an inflammatory vicious circle. The high amount of proteolytic enzymes results in local inflammation of the gland, with ongoing damage to the pancreas [9, 10] and release of proinflammatory cytokines interleukin (IL)-1, IL-6, and IL-8 and systemic mediators such as tumor necrosis factor alpha (TNF-α) inducing a systemic inflammatory state. This process is often mild and self-limiting, but the progression of inflammation can lead to a severe disease graved by local complications, systemic inflammatory response syndrome (SIRS), MODS, and death [11]. In the severe form of AP, the systemic inflammation induces macrovascular and microvascular alterations, leading to an increased capillary permeability. This in turn induces an interstitial fluid accumulation with a decreased circulating volume, causing functional hypovolemia with renal hypoperfusion and acute kidney injury. Pancreatic microvascular alterations include shunts, vasoconstriction, increased permeability, edema, and increased leukocyte adhesion [12]. Pancreatic tissue has been shown to be very sensitive to hypoxemia and ischemia with rapid progression to necrosis if local circulation is compromised [13]. Currently the most effective treatment measure is aggressive fluid resuscitation in order to restore an adequate circulating volume. However, pancreatic circulation during AP has been proven to be indirectly correlated to cardiac output. This indirect correlation is due to the microvasculatory changes happening during AP [12], thus explaining why even a sufficient systemic perfusion is sometimes not sufficient to provide an adequate local perfusion and prevent necrosis.

Two phases have been described in the development of complications during severe AP: early phase and late phase. The early phase takes place during the first 10 days and is characterized by SIRS and/or MODS. During this initial period, a marked hypovolemia is observed, accentuated by increased capillary permeability, ascites, and ileus, leading in combination with septic complications to renal dysfunction. Almost half of the patients with severe AP develop pulmonary complications, ranging from mild hypoxemia to acute respiratory distress syndrome [10].

The late phase has been associated with the development of infections [14] such as pneumonia and bacteremia [3, 15, 16] and is characterized by local complications (i.e., pancreatic and peripancreatic necrosis, peripancreatic fluid collections, pseudocysts, infection of necrotic tissue) [3]. Moreover, hypovolemia and hypoxemia due to MODS diminish the oxygen supply to the pancreas and promote the development of necrosis. Pancreatic necrosis is a severe complication because of its secondary infection risk. Infected necrosis by gut-derived bacteria is responsible for up to 80 % of mortality in AP [17]. Sterile pancreatic necrosis with bacteremia is associated with a high infection risk, especially when the gastrointestinal barrier is compromised [18].

### Thoracic epidural analgesia in sepsis

Sympathetic blocks induced by TEA have been shown to induce peripheral and splanchnic vasodilatation, improve gut mucosal perfusion, delay intestinal acidosis in hypoxia, and increase intramucosal pH in patients with peritonitis [6]. However, TEA can also lead to functional hypovolemia and secondary hypotension, which is mainly seen in the presence of extensive thoracolumbar block [19]. In the context of sepsis or SIRS, when cardiovascular dysfunction leads to a reduced vascular tone, TEA could be deleterious. Daudel et al., investigating the role of TEA during an experimental model of endotoxemia, showed that an epidural block of T2–T10 did not impair cardiopulmonary hemodynamic conditions and global oxygen transport, beyond the changes induced by the endotoxin itself. Two hypotheses were formulated to explain the absence of hypotension in this localized block: either the localized vasodilatation was sufficiently compensated by vasoconstriction in nonblocked regions; or endotoxemia had already induced a maximal vasodilatation response, preventing a further decrease secondary to sympathetic blockade [19].

The bowel mucosa has a particular vascular architecture and is a very vulnerable tissue to hypoperfusion and hypoxia, requiring extensive amounts of oxygen to maintain functional integrity. In sepsis, microvascular derecruitment of vascular beds occurs, thus compromising the intestinal mucosal barrier and leading to translocation of bacteria and toxins with secondary bacteremia. Daudel et al. [20] also investigated the effects of TEA on gut mucosal microcirculation in septic rats and showed that TEA increased the density of capillary networks and improved the quality of gut mucosal perfusion.

The liver, as a main regulator of homeostasis to injury, sepsis, and inflammation, also represents a target of TEA. During AP, the activation of the sympathetic system leads to an increase of catecholamines, inducing activation of hepatocytes, Kupffer cells, and neutrophilic granulocytes. The resulting cytokine increase of IL-6, TNF-α, and Fas ligand (FasL) induces systemic inflammation and direct effects on the liver, causing local hepatic damages and hepatocyte apoptosis [21]. These hepatic alterations are worsened by α-adrenergic and β-adrenergic receptor stimulation, which can induce intrahepatic inflammation and liver injury in healthy mice without any additional stimuli. In severe sepsis, liver failure is associated with increased mortality and length of hospital stay. Hepatic sympathetic mechanical denervation has been shown to reduce liver injury during stressful situations [22]. In 2009, Freise et al. investigated the role of TEA on liver function in sepsis in rats. They showed a pathological increase in sinusoidal blood flow in the AP group, restored to control values with TEA. No significant changes were noted in mean blood pressure, cardiac output, hepatic enzymes, and TNF-α in animals with TEA [22].

### Epidural analgesia in animal acute pancreatitis

Animal studies focusing on the role of TEA in AP are summarized in Table 1. The main findings include mortality reduction in rats and pigs, reduced metabolic acidosis, reduced hepatocyte apoptosis and sinusoid vasoconstriction, and improvement of pancreatic and ileal perfusion. A specific discussion on these articles follows.

**Table 1:** Animal studies focusing on the role of thoracic epidural analgesia in acute pancreatitis

First author and reference	Year	Subjects	Number	Groups	Epidural analgesia	Main measures	Findings	Comments
Demirag [23]	2006	Rats	19	Three groups: 1) AP [9] 2) TEA [4] 3) AP + TEA [6]	Catheter positioned between T7 and T9. Bupivacaine 0.4 % 20 μl/h	Hemodynamic and biological parameters Arterial blood gases Laser Doppler flowmetry of pancreatic microcirculation Histopathological analysis	- TEA induced a strong increase in pancreatic microcirculation compared with AP group - TEA reduced the severity of metabolic acidosis - Histopathological analysis showed that TEA reduced acinar cells, without reaching statistical evidence	Three different groups Histological analysis Reproducibility of the procedure
Lauer [24]	2007	Rats	21	Three groups: 1) AP [7] 2) AP + TEA [7] 3) Sham^a^ + TEA [7]	Catheter insertion in L3– L4, advanced to T6. Bupivacaine 0.5 % 15 μl/h	In-vivo and in-vitro analysis Hemodynamic and biological parameters Arterial blood gases Receptor-independent vasoconstriction Receptor-dependent and independent vasoconstriction by angiotensin and bradykinin	- AP group had more severe metabolic and lactate acidosis compared with rats benefitting from TEA - TEA induced a better arterial oxygenation and higher mean arterial pressure compared with AP - TEA induced a better hypoxic vasoconstriction response associated with a reduction of exhaled nitric oxide, showing less smooth muscle cell dysfunction compared with AP - TEA induced a better receptor sensitivity to angiotensin II and bradykinin compared with AP	Three different groups In-vivo and in-vitro analysis Reproducibility of the procedure
Freise [26]	2009	Rats	28 + 22	Four groups: 1) Sham^a^ + NaCl [7] 2) Sham^a^ + TEA (7 + 8) 3) AP + NaCl (7 + 7) 4) AP + TEA (7 + 7)	Catheter insertion in L3– L4, advanced to T6. Bupivacaine 0.5 % 15 μl/h	Hemodynamic and biological parameters Arterial blood gases Intravital microscopy for sinusoid perfusion and diameter FasL expression measures Histopathological analysis	- TEA reduced overall apoptosis and hepatocyte apoptosis compared with AP - TEA prevented sinusoid vasoconstriction, but did not influence loss of sinusoids and sinusoidal perfusion compared with AP - TEA did not induce significant changes in hemodynamic parameters, FasL expression, and arterial blood gases	Four different groups Blinded investigators and pathologist Large sample with 22 rats added for histopathological analysis and FasL measures Reproducibility of the procedure
Freise [18]	2006	Rats	28	Four groups: 1) Sham^a^ + NaCl [7] 2) AP + NaCl [7] 3) AP + TEA [7] 4) AP + TEA 7 hours after AP induction	Catheter insertion in L3– L4, advanced to T6. Bupivacaine 0.5 % 15 μl/h	Intravital microscopy of ileal mucosa Hemodynamic and biological parameters Arterial blood gases Histopathological analysis	- TEA increased survival at 7 days from 33 to 73 % compared with AP group - TEA did not induce more hypotension compared with AP group - A reduction of 50 % of the ileal arteriolar perfusion was observed in the AP group, restored to normal in the AP + TEA and in delayed TEA groups - TEA reduced lactate concentration in the AP + TEA and delayed TEA group compared with AP - TEA reduced histologic injury compared with AP alone, without reaching statistical evidence - TEA induced a diminution of IL-6 plasmatic concentration compared with AP	7 days of follow-up Data on mortality Four different groups, one with delayed TEA, closer to clinical situation Blinding of investigators and pathologists Reproducibility of the procedure
Bachmann [27]	2013	Pigs	34	Two groups: 1) AP + TEA [13] 2) AP alone [21]	Catheter positioned between T7 and T8. Bupivacaine 0.5 % bolus + continuous 4 ml/h	Hemodynamic and biological parameters, arterial blood gases Laser Doppler flow measures of pancreas microcirculation All of the experiment was realized in ICU settings with hemodynamic monitoring and automated infusion system	- TEA improved overall survival at 7 days, from 29 % in AP group to 82 % in the group receiving TEA - TEA improved microcirculation and tissue oxygenation of the pancreas - TEA reduced histopathological pancreatic lesion compared with AP	7 days of follow-up Data on mortality Experiment on big animals, allowing controlled hemodynamic conditions such as in ICU settings Verification of TEA spread by epidurogram Blinded pathologist Reproducibility of the procedure

^a^Sham defines a group with no induction of acute pancreatitis

*TEA* thoracic epidural analgesia, *AP* acute pancreatitis, *FasL* Fas ligand, *IL* interleukin

In 2006, Demirag et al. [23] showed for the first time the benefit of TEA on the severity of AP in rats. The authors evaluated the impact of TEA during AP by measuring the pancreatic microcirculatory flow and arterial blood gases, and completed the experiment with histological analyses to investigate the development of pancreatic necrosis. TEA partially restored the severe reduction in pancreatic blood flow occurring after AP induction, and reduced the severity of metabolic acidosis. The histopathology, performed less than 5 hours after AP induction, showed extensive edema and tissue necrosis in the AP group with less extensive damage in the TEA group without reaching statistical significance [23].

To investigate the pulmonary repercussions of TEA during AP, Lauer et al. [24] developed an in-vivo and in-vitro model of AP and TEA in rats. They showed many effects of AP on the hemodynamic conditions and lung function: pulmonary edema, reduced oxygenation, development of metabolic acidosis, increased myeloperoxidase activity (marker of neutrophil infiltration in the lung tissue), and severe endothelial dysfunction. They showed a beneficial role of TEA with following results: the AP + TEA group compared with the AP group had better PaO_2_ and higher mean arterial pressure and developed almost no metabolic acidosis, whereas the AP group developed lactate acidosis. AP also severely impaired lung vasoadaptative mechanisms such as hypoxic pulmonary vasoconstriction (HPV), an important adaptative mechanism depending on the integrity of vascular smooth muscle cells, redirecting blood flow from hypoxic to normoxic regions, and thus optimizing the ventilation/perfusion ratio [24]. One of the hypotheses for HPV impairment is an increased production of nitric oxide (NO) in pancreatitis. In the context of endothelial dysfunction, increased NO production, normally known to be anti-inflammatory and vasodilatative, is thought to hinder protective vasoconstriction and alter HPV. The TEA group showed lower levels of exhaled NO, associated with a better HPV regulation. In the context of endothelial dysfunction, improvement of HPV by NO synthase inhibitors is described in the literature [25]. They also investigated receptor-dependent pulmonary vasoconstriction and found an increased vasoreactivity to Angiotensin II and to bradykinin in the TEA group, showing better receptor-dependent vasoconstriction. It is noteworthy here to signal that bradykinin normally only induces vasoconstriction when the endothelium is deficient. The authors discussed this issue with the hypothesis that TEA might improve receptor function itself. All of these findings suggest an improved lung adaptation mechanism to inflammatory injury by TEA.

Hepatic effects of TEA during AP were investigated in a study published by Freise et al. [26] where they measured hepatic perfusion by intravital microscopy and FasL concentration and performed histopathological analysis. They showed a reduction in sinusoidal vasoconstriction, without any influence on loss of sinusoids and sinusoidal perfusion. TEA reduced overall apoptosis and hepatocyte apoptosis significantly. No significant changes in FasL expression were noted. These results suggest that TEA plays a role in hepatic dysfunction occurring during AP by reducing hepatocyte apoptosis [26].

Freise et al. [18] conducted another study in rats randomized into four groups (TEA without AP, AP, AP + TEA, and AP + TEA delayed 7 hours after AP induction), focusing on survival and pancreatic perfusion. They showed a decrease of 50 % of the ileal mucosal arteriolar blood flow after induction of AP, which was restored to normal in the TEA group and the delayed TEA group. More impressively, they showed an increased 7-day survival rate of 73 % in the AP + TEA group as compared with 33 % in the AP group (*P* < 0.05). They also measured the plasmatic concentration of IL-6, and showed a significant reduced serum concentration in the TEA and the delayed TEA groups compared with AP [18]. This finding is noteworthy since IL-6 contributes to the disruption of the gastrointestinal barrier, thus promoting bacterial translocation and endotoxemia, and increasing the risk of pancreatic necrosis infection.

Bachmann et al. [27] reported an experimental study in pigs where one group of animals underwent AP and TEA, while control animals underwent AP only. During the first 6 hours, all animals had controlled hemodynamic conditions with fluid administration or catecholamine infusion if needed. Tissue perfusion and oxygenation was measured by surgical laparotomy. After AP induction, blood flow and tissue oxygenation diminished rapidly in both groups, but animals receiving TEA maintained a significantly higher perfusion and tissue oxygenation when compared with the control animals. Histopathological analysis revealed significantly less severe pancreatic lesions in the group benefiting from TEA. The main outcome of this study was the 7-day survival, showing 82 % of animals alive in the TEA group compared with only 29 % in the control group (*P* < 0.05) [27]. A main force of this study is the use of large animals, which allowed the authors to monitor animals in conditions similar to the intensive care setting. These results indicate the crucial role of TEA on mortality in a large animal model of AP.

We have discussed many positive aspects of local anesthetics (LA) administered in an epidural way in animals experiencing AP. There exists evidence that intravenous LA play an anti-inflammatory role in the lung, the endothelial cells, and the gastrointestinal tract [28]. This is interesting since a considerable resorption of LA occurs in the systemic circulation [29]. Cassuto et al. [30] in 2006 reviewed the literature concerning the anti-inflammatory role of LA. The LA from the amino-amide class, such as lidocaine and bupivacaine, have a dose-dependent anti-inflammatory action when injected intravenously. They reduce leukocyte adhesion and migration, phagocytosis, and oxygen free radical production. They also reduce the synthesis of inflammatory mediators by inhibition of various cell membrane-associated enzymes and inhibit cytokines release such as IL-1, TNF-α, and IL-8 [30]. LA also possess a bactericidal action mostly related to their concentration and partly to their structure. Ropivacaine appears to be an exception, showing less or no anti-inflammatory and bactericidal activity. These results suggest that TEA might also have an indirect anti-inflammatory action by its systemic resorption.

### Epidural analgesia in human acute pancreatitis – safety and efficacy

The impact of TEA on the human physiology has been studied extensively, but is still not fully understood. Most of the knowledge we have comes from experimental studies. Human studies focusing on the role of TEA in AP are summarized in Table 2. The main findings include the safety of use in patients presenting severe sepsis and septic shock and an improvement in pancreatic perfusion. Side-effects (hypotension, epidural abscess) and the protocol of epidural analgesia are presented. A specific discussion concerning these articles follows.

**Table 2:** Human studies focusing on the role of thoracic epidural analgesia in acute pancreatitis

First author and reference	Year	Number	Type of study	Epidural analgesia	Measures	Findings	Strength of the study
Bernhardt [31]	2002	121	Prospective observational study	Catheters were placed in the thoracolumbar region, varying from T8 to L3, most of them being placed between T10 and L1 70 % were thoracic blocks and 30 % were lumbar blocks Epidural analgesia was managed with boli of 3–5 ml of bupivacaine 0.25 % every 4–6 hours. No continuous administration	Safety of procedure Number of doses given Mortality Pain Number of surgeries needed Days of artificial ventilation Biological parameters	- The median epidural block length was 4.2 days - TEA was well tolerated and considered safe even in severe patients - Catheter-associated hypotension occurred in 12 % of the cases, manageable without complication with fluid replacement and amines - Excellent analgesia was achieved in 72 % of observation days without additional drugs - Normalization of pancreatic enzymes occurred sooner in patients with early placement of catheter -13 % of patients required artificial ventilation (mean 12 days) - 24 % of patients had accidental removal of catheter: 7.4 % had three catheters placed, 3.3 % had four catheters placed, without any infectious complications - Average duration of ICU was 12.4 days	Large sample, representative of general population (mean 53.2 years, extremes 15–87) Precise follow-up of doses needed to reach sufficient analgesia
Jabaudon [32]	2015	121	Prospective observational multicenter study	Catheters were placed in the thoracolumbar region: 89 % were thoracic blocks and 11 % were lumbar blocks Each center had its own epidural protocol: 26 % used levobupivacaine and 74 % used ropivacaine Local anesthetics were always combined with sufentanil	Safety of procedure in severe patients Mortality Reason for initiating TEA Sepsis status ICU standard measures	- The mean epidural block length was 11 days - 38 patients (31 %) had acute pancreatitis - 8 % of patients presented catheter-associated hypotension, 2.5 % required punctual administration of vasopressin during the catheter placement - 60 % of patients experienced sepsis, 42 % severe sepsis, 22 % septic shock and showed good tolerance to TEA - 65 % of patients required mechanical ventilation during the ICU stay - 17 % had accidental removal of catheter - one case of epidural abscess	Large sample, but only 38 patients experiencing AP Multicenter study Long epidural duration Good tolerance in severe disease
Sadowski [33]	2015	35	Randomized control trial Group 1: AP + TEA [13] Group 2: AP alone [22]	All catheters were placed at the thoracic level between T6 and T9, reaching a T4–T12 sensitive block Epidural analgesia was managed on a patient-controlled protocol, with continuous infusion of bupivacaine 0.1 % + fentanyl 2 μg/ml 6–15 ml/h + boli of 3–5 ml every 30–60 min	Safety of TEA in severe AP patients CT perfusion protocol on admission and 72 h (>20 % difference considered significant) Pain measure, length of hospital stay Use of antibiotics Admission to ICU Patient demographics, comorbidities and etiology of AP	- The median epidural block length was 5.7 days - More patients increased their pancreatic perfusion in the group benefitting from TEA compared with AP (43 % vs 7 % respectively) - TEA helped reduce visual analog pain score compared with AP - No significant differences were noted in ICU admission - Less patients in the TEA group required artificial ventilation compared with AP, without reaching statistical evidence (7.7 % vs 27.3 %, *P* = 0.22) - No differences were observed in locoregional and systemic complications - No length of stay difference	Randomized control trial Study conducted on patients experiencing severe disease Blinded radiologist

*TEA* thoracic epidural analgesia, *AP* acute pancreatitis

Only a few studies have investigated the role of TEA in humans with AP, and most of them focused on the safety of use more than on the changes in prognosis. Bernhardt et al. [31] analyzed the safety of TEA during AP in a group of 121 patients. The authors could show that epidural injection of LA was well tolerated, even in patients with marginal cardiovascular stability. No neurological or infectious complications were reported. Excellent analgesia was achieved in 72 % of the observation days by TEA only, without use of systemic analgesic substances. Only 8 % of LA injections were associated with hemodynamic changes requiring use of sympathomimetic drugs. The conclusion of the study was that TEA is a safe procedure in patients experiencing AP even in its severe form, and that cardiovascular instability should not be considered as a contraindication of TEA. However, septic shock situations should be approached with caution. This study did not investigate the prognosis of patients, but postulated an improvement in early mobilization, with fewer embolic and respiratory complications [31].

To assess the feasibility, practice, and safety of epidural analgesia in the ICU, Jabaudon et al. [32] published in 2015 a prospective multicenter observational study. They included 121 patients, of which 38 (31 %) had AP. Other main causes of inclusion were major surgery (42 %) and trauma (14 %). Most of the patients presented organ failure on admission. Criteria for sepsis, severe sepsis, and septic shock were present in 60 %, 42 %, and 22 % of the patients respectively. The mean length of stay of the epidural catheter was 11 days with extreme values ranging from 3 to 38 days.

The observed adverse effects of TEA were the following: 8 % of the patients presented a catheter placement-related hypotension which responded adequately to intravenous fluid administration of 250–500 ml; and 2.5 % required vasopressor therapy during the catheter placement procedure in addition to the fluid resuscitation. Furthermore, one case (0.8 %) of epidural abscess was observed, with an incidence rate higher than reported previously in anesthesia and perioperative medicine [5]. The authors admitted that this abscess could have reasonably been avoided with a strict protocol for catheter insertion and management. No spinal or epidural hematomas were reported, despite the accidental removal rate of 17 %. Their study suggested safety of use of TEA during organ failure, such as that appearing in AP, with the requirements of a close follow-up looking carefully for signs of infections and a timely removal of catheters in patients with suspected or known untreated bacteremia [32].

Another randomized prospective pilot study was conducted by Sadowski et al. [33] on 35 patients with severe forms of AP. Patients were randomized into two groups, one receiving TEA and the other systemic analgesia. The primary outcome was safety of TEA in AP patients. TEA was placed for a median time of 5.7 days, and no hemodynamic or infectious complications occurred. Pancreatic perfusion was evaluated by two injected CT scans at admission and 72 hours later. A significant variation of pancreatic perfusion was defined as a difference of at least 20 % between the two time points. Improved pancreas perfusion (20 % increase or more) was observed for 43 % patients in the TEA group vs 7 % in the control group. TEA allowed better pain management, assessed by a visual analog pain scale every 8 hours. Hospital length of stay and mortality (0 % in both groups) were not statistically different. This pilot study showed that TEA increased arterial pancreas perfusion and was safe for use in patients with severe AP [33].

All of these recent studies suggest that the use of TEA during AP is safe, and can possibly induce an improvement in pancreatic perfusion. Hypotension should not be retained as a contraindication for TEA during AP, since it is manageable by fluid resuscitation or pharmacological interventions.

## Discussion

We have shown throughout this review the existence of increasing evidence that TEA plays a regulatory role in many organ dysfunctions during severe AP, such as that of the pancreas itself, the gut, the liver, and the lungs. Indeed, TEA improves intestinal blood flow, pancreatic microcirculation, and lung and liver function, reduces the extent of pancreatic necrosis, and helps preserve the gastrointestinal barrier during AP. All of these factors are crucial in the development of AP complications. Animal models also showed an important mortality reduction in animals receiving TEA [18, 27]. All these findings suggest that TEA is an efficient approach to reduce the proinflammatory state and improve the outcome of the disease.

Many questions remain concerning the clinical application of TEA: where and when should the catheter be placed and how long should it stay in place? What block extension is required? Should only patients with severe disease benefit from TEA or is there also a benefit for milder disease? What type of anesthetics should be used? During the following discussion, we aim to discuss these practical aspects one by one.

To begin this discussion, it is important to say that TEA is already used by many clinicians in the context of AP, mainly for analgesic purposes when conventional analgesic therapy is insufficient. To determine where to place the block, it is relevant to know that pancreas sympathetic afferent innervations originate in T6–L2 [34]. Knowing that catecholamine release, one of the main factors contributing to maintaining blood pressure, occurs by stimulation of the adrenal medulla innervated by T5–L1 [35], hypotension is one of the awaited side-effects from pancreatic regional sympathetic blockade. In most of the human studies we reviewed, the block was placed either in a low thoracic position or in an apical lumbar position (Table 2); and in animal experiments, all of the catheters were positioned in a low thoracic position (Table 1), with an incidence of hypotension around 10 %. We could not find pertinent articles concerning extended blocks to the cardiac region during AP; however, we consider that extending this block to the cardiopulmonary region might diminish the cardiac response to hypotension and increase the frequency and severity of hypotension. Based on existing experimental studies, a low thoracic block or apical lumbar block should be used, with a preference for the low thoracic block to minimize the block extension with regard to the risk of hypotension.

Combined epidural analgesia (CEA) is another approach to reduce the doses of LA and improve analgesia. Jabaudon et al. [32] and Sadowski et al. [33] used sufentanil and fentanyl respectively, whereas Bernhardt et al. [31] used bupivacaine only, with a satisfactory analgesia in all studies. In a randomized, double-blind trial focusing on postthoratocomy pain, Grider et al. [36] showed that a combination of hydromorphone with bupivacaine vs bupivacaine alone resulted in better analgesia with smaller doses and a reduced hypotension rate in the CEA group. Another prospective randomized trial realized in the same context by Tuncel et al. [37] also showed superior pain relief by a combination of sufentanil with ropivacaine vs ropivacaine alone, without significant difference in the hypotension rate. CEA should therefore be used to minimize the risk of hypotension.

When comparing animal models with human AP pathophysiology, it is important to consider that experimental studies mimic a hyperacute model of AP, with TEA effective within minutes or hours following the induction of AP. This situation is not realistic for clinical practice, when TEA could not be performed earlier than hours or days after induction. The remaining question is whether the TEA benefits will continue if initiated within these delays, and which kind of patients could benefit from this procedure? Bernhardt et al. [31] observed that the earlier TEA was started in humans, the faster pancreatic specific enzymes decreased in the serum of patients. This beneficial effect was still observed after several days, suggesting that TEA maintains a beneficial role even if not installed immediately [28]. To consider which patients should benefit from TEA, it is crucial to identify patients at high risk of developing a severe disease, since the purpose of TEA placement is to avoid further complications and to minimize already present complications. In the last revision of the Atlanta Classification [38], the Marshall score [39] – based on respiratory, renal, and cardiovascular dysfunction – was defined as a simple, efficient, and universal score to identify patients experiencing severe disease [38]. We propose to use the Marshall score as an aid for physicians to identify rapidly severe cases of AP, which should then benefit from TEA initiated as early as possible.

Another unresolved issue is the optimal length of treatment, maximizing potential benefits and minimizing infectious complications. Bernhardt et al. [31] and Sadowski et al. [33] observed good clinical tolerance and no infectious complications with a mean in-place catheter duration of 4.2 days and 5.7 days respectively, whereas Jabaudon et al. [32] also observed good tolerance with a mean duration of 11 days, but with one case of epidural abscess [32]. TEA management is not clearly standardized and several protocols exist with regard to avoiding procedure-related complications. We should keep in mind that epidural abscess is the most feared and severe complication of TEA, but is rare with adequate management. For example, known untreated bacteremia should be a sufficient reason to remove the catheter. Concerning catheter dislocation that happened in 24 % and 17 % patients in the studies of Bernhardt et al. [31] and Jabaudon et al. [32] respectively, the catheter was safely replaced in patients showing no signs of local infection. Moreover, Jabaudon et al. showed that 74 % of the patients required only one catheter placement, whereas two catheters were placed in 15 %, three catheters in 10 %, and four catheters in 2 %, with a mean catheter colonization of 22 %. Some centers propose to change the catheter every 7–10 days to avoid infection risks. To maximize the effects of TEA, we propose that the catheter should stay in place as long as it is required for pain management, since there exists no simple way to test directly the induced sympathectomy. Catheter replacement should be discussed if catheters stay in place for a period exceeding 7–10 days or if they are accidentally removed. A close follow-up searching for local and systemic signs of untreated infection is required, and the catheter should be removed as soon as serious infectious risks are detected.

## Conclusion

There is a real need for new therapeutic approaches for the treatment of severe AP, for which the mortality is still high for severe disease. The growing understanding of the disease has proven that microcirculation plays a crucial role in the development of pancreatic necrosis. TEA appears to be a promising complementary approach, acting on microcirculation and many organs dysfunctioning in AP which are not influenced by conventional treatment. TEA appears as a safe and effective procedure in humans experiencing severe AP, allowing better analgesia and potential benefits. Close follow-up of patients benefiting from TEA is required to manage side-effects such as hypotension and avoid epidural abscess. Multicenter randomized trials are becoming mandatory to investigate the effects of TEA on morbidity and mortality in humans undergoing a severe form of AP.

## Abbreviations

AP: acute pancreatitis
CEA: combined epidural analgesia
FasL: Fas ligand
HPV: hypoxic pulmonary vasoconstriction
IL: interleukin
LA: local anesthetics
MODS: multiorgan dysfunction syndrome
NO: nitric oxide
SIRS: systemic inflammatory response syndrome
TEA: thoracic epidural analgesia
TNF- α: tumor necrosis factor alpha
